# Assessing eosinophilic cationic protein as a biomarker for monitoring patients with eosinophilic esophagitis treated with specific exclusion diets

**DOI:** 10.1186/s40413-017-0143-6

**Published:** 2017-03-23

**Authors:** Joan Doménech Witek, Vicente Jover Cerdà, Vicente Gil Guillén, Juan Bautista Doménech Clar, Ramón Rodríguez Pacheco

**Affiliations:** 1 Hospital General de Elda, Sección de Alergología. H. Elda, Carretera de Sax, 03600 Elda, Alicante Spain; 2 Universidad Miguel Hernandez de Elche, Alicante, Spain; 3 Hospital Universitario Dr. Peset, València, Spain

**Keywords:** Eosinophilic esophagitis, Eosinophil cationic protein, Specific diet

## Abstract

**Background:**

Eosinophilic esophagitis (EoE) is a complex pathology. Attempts have been made in order to relate EoE with the intake of certain food. The problem is to establish which foods are really involved in the pathophysiology of this condition and to objectify a reliable inflammation biomarker for the follow-up of patients undergoing pharmacological treatment and/or diets. Our aim is to assess the food sensitization profile of patients with objective diagnosis of EoE and objectify the utility of ECP as an inflammation biomarker for the follow-up of patients with EoE treated with specific diets, based on the hypothesis that we will observe a decrease and clinical improvement after maintenance of these diets.

**Methods:**

A total of 19 subjects were included between 1 January 2012 and 30 June 2015. Diets based on allergy testing were established. Prior to the initiation of the diets, baseline ECP was determined. Appointments were arranged for the patients between 4 and 6 months later to assess the clinical response to the specific diets and to request a second blood sample for blood counts and serum ECP levels to compare with the previous baseline.

**Results:**

19 patients diagnosed with EoE (12 males and 7 females) between January 2012 and June 2015, aged between 17 and 68 (33.52; SD 13.67 years), were included consecutively, 15 of whom showed optimum response to specific diets based on allergy testing. A statistically significant difference ECP decrease was observed in our patients.

**Discussion:**

Until now most of the studies previously published in reference to the use of ECP as a biomarker for monitoring patients on treatment with diets show consistent but insignificant decreases in ECP levels. However, ECP seems to be a good marker of inflammation if the determinations are performed avoiding confounding factors.

**Conclusion:**

The serial determination of ECP is useful when monitoring patients with EoE treated with specific diets.

## Background

Eosinophilic esophagitis (EoE) can be caused by immunoglobulin E (IgE)-mediated, non-IgE-mediated or mixed food sensitization mechanisms. EoE is characterized by the presence of eosinophil infiltration in the oesophagus, with peripheral eosinophilia observed in ~50% of cases. EoE has no pathognomonic clinical manifestations, is more common in men, and can manifest at any age [1]. In children, the most frequent manifestations are feeding difficulties, growth disorders, vomiting and abdominal pain. In adults, the most frequent manifestations are dysphagia, gastroesophageal reflux, abdominal pain and impaction episodes of the alimentary bolus. Furthermore, an important issue is the lack of correlation between symptom intensity and tissue inflammation.

In patients with dysphagia, endoscopy and anatomo-pathology testing can confirm cases of EoE in 15 to 35% of cases [2]. However, reliable inflammatory biomarkers need to be identified to limit the number of invasive endoscopies needed for the adequate monitoring of EoE patients.

It is now acknowledged that EoE requires interdisciplinary management by gastroenterologists, allergists and anatomical-pathologists. Objective sensitization to food allergens is fundamental to establishing diets that avoid the evolution of oesophagitis to fibrosis and the development of oesophageal stricture states that can trigger serious complications, including impaction and possibly oesophageal perforation.

Eosinophilic cationic protein (ECP) is a highly cationic protein with numerous biological functions and cytotoxic effects, and it is stored by eosinophils and accumulated by neutrophils. ECP levels can be measured in many biological fluids, and though it is most commonly used to study asthma [3], ECP may also be a useful marker for monitoring EoE. In particular, better testing methods are needed in the context of food avoidance diets, which are often complex and involve multiple testing steps after the sequential reintroduction of each type of food. Previous studies have described short patient series in which ECP appears to be a useful biomarker (determined in blood or faeces). However, further studies are needed involving larger sample sizes to determine the usefulness of ECP as a biomarker, not only for initial avoidance but also for the reintroduction of each kind of food and subsequent monitoring of EoE patients.

The aim of this study was to better characterize a number of previously identified biomarkers, including IgE [4] and ECP [5] levels. We seek to establish the usefulness of ECP for the monitoring of patients with EoE under treatment with specific exclusion diets. Considering that ECP levels can vary due to many factors, particularly those associated with respiratory pathologies or poorly controlled diets, one of our priorities was to eliminate these confounding factors prior to characterizing this biomarker as a patient monitoring tool.

## Methods

### Study design

To determine the usefulness of ECP as a biomarker, the following hypothesis was suggested based on our clinical experience: Serum ECP levels will significantly decrease after recommended specific diets have been established following objective food sensitization by means of skin tests or specific IgE. Furthermore, we expect the objective decrease to correlate with clinical improvements and possibly with the cessation of symptomatic treatments with proton-pump inhibitors or swallowed/inhaled corticosteroids.

The study included adult and adolescent patients referred by a digestive medicine specialist with an objective diagnosis of EoE, which was based on currently accepted metrics (15 or more eosinophils per oesophageal mucosal biopsy field) [1]. A total of 19 subjects were included between 1 January 2012 and 30 June 2015. During the initial appointment, relevant medical history information was gathered, and skin tests were performed with complete batteries of standard aeroallergens (23 items) and food allergens (62 items) (prick tests were performed using standardized allergen extracts from Bial Aristegui® Laboratories). Patients were then referred to a hospital laboratory for a blood sample to be taken to establish IgE levels specific to food, blood count and serum ECP (baseline prior to establishing relevant specific diets). Patients were informed that the blood sample had to be taken when the subject had no respiratory symptoms compatible with rhinitis or asthma and no active infection; the blood sample collection took place outside the relevant aggravation period in each case (in relation to aero-allergen sensitization). Appointments were arranged for all patients to discuss the results of their tests and to recommend specific diets. We indicated to our patients the avoidance of food whose sensitization had been objectified by skin tests, specific IgE levels higher than 0.35 kUA/L as well as foods with cross-reactivity with pollen to which the patients were sensitized (mainly fruits and the solanaceae family). After the diets had been established, appointments were arranged for the patients between 4 and 6 months later to assess the clinical response to the specific diets and to request a second blood sample for blood counts and serum ECP levels to compare with the previous baseline. The characteristics of patients included in the study with analytical values and endoscopic findings are summarized in Table 1.

**Table 1:** Characteristics of patients included in the study with analytical values and endoscopic findings

Number of patients	19
Men/Women	12/7 (63.15%/36.85%)
Age	33.52 ± 13.67
Asthma	4 (21%)
Rhinoconjunctivitis	12 (63.15%)
Atopic dermatitis	5 (26.3%)
Aeroallergens	13 (68.4%)
Food allergies	16 (84.2%)
Overall clinical response to diet	
Improvement	15 (79%)
No response	1 (5.22%)
Not included	3 (15.78%)
Endoscopic findings	
Eosinophil in biopsies (n/hpf)	22,06 ± 5,34
Inflammatory phenotype	9 (47.36%)
Stenosing phenotipe	4 (21%)
Mixed phenotype	6 (31,64%)
Analytical values	
Total IgE	255,53 ± 272,26
ECP Pre diet (μg/mL)	34,25 ± 26,37
Eosinophils in peripheral blood	343,33 ± 177,83

Patients who did not follow the criteria of providing blood samples during a period without respiratory symptoms or outside the aggravation period (the majority of samples were taken in August, December and January) were excluded. Furthermore, patients who had previously started diets prior to consultation were not included in the study.

Patients were told to continue with their symptomatic treatments as directed by their gastroenterologists for 2 to 3 months and then to stop the treatment and continue with the diets alone if they began to see improvements.

### Histological and endoscopic studies

Endoscopic procedures were performed by an experienced gastroenterologist using a flexible 8.5 mm gastroscope with a 2.8 mm work channel (EG-530 FP, Fujinon, Fujifilm Corporation. Tokyo, Japan). Biopsies were performed using conventional forceps (Radial JawTM 4 Boston Scientific, Proparck, Costa Rica). No complications were observed as a result of the endoscopic procedures or biopsies performed. The histological study was performed only to have a confirmed diagnosis of EoE, not carrying out a control study after the maintenance of the indicated diets.

Biopsy specimens were sent to the Pathology Department, where they were formalin-fixed and paraffin-embedded. Serial sections were then performed using a microtome and stained with eosin haematoxylin. EoE was diagnosed in cases with 15 eosinophils/hpf or more in at least one high-power field (HPF).

### Analysis

Comparisons were performed using non-parametric tests of paired samples. Each individual was compared to their baseline measurements. Continuous variables (ECP) are expressed in mean ± SD values, and the Wilcoxon rank test was used to contrast hypotheses regarding continuous variables for pre- and post-diet. Graphs show box plots with median and interquartile ranges.

## Results

### Epidemiological data

Nineteen patients diagnosed with EoE (12 males and 7 females) between January 2012 and June 2015, aged between 17 and 68 (33.52; SD 13.67 years), were included consecutively. The epidemiological variables are summarized in Table 1.

### Clinical data

Most of our patients presented food sensitization, and we observed atypical food allergies, being the most prevalent allergy to nuts, legumes, gluten and milk. Objectified food sensitizations are summarized in Table 2. The most common atopic feature was rhino-conjunctivitis with some cases of asthma and atopic dermatitis. The phenotypic diversity of the patients studied, from patients with multiple food and environmental sensitizations to patients with no atopic trait or associated respiratory pathology, was noteworthy. We also encountered a number of cases in which patients said their digestive symptoms were worse during aggravation periods of their respiratory symptoms due to pollen exposure.

**Table 2:** Food sensitization objectified by percentage of each kind of food

Nuts	19%
Rice/Vegetables	19%
Gluten	18%
Milk	13%
Peach	11%
Egg	9%
Banana/Kiwi	8%
Fish	2%
Meat Anisakis Solanaceae Cocoa Other fruits	<1%

### Analytical values

**Fig. 1:**
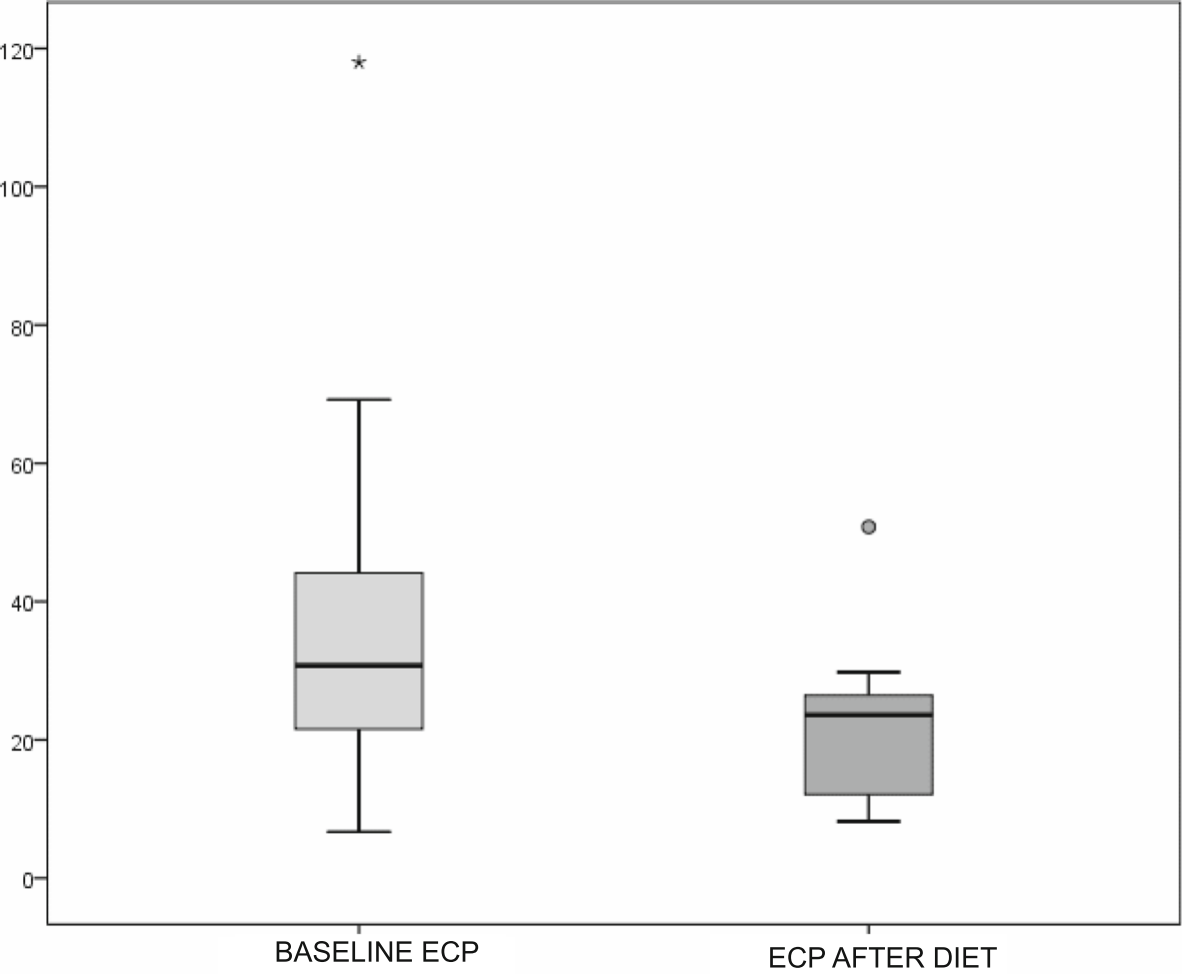
Significant differences between baseline ECP levels (34.25 (μg/mL) ± 26.37) and ECP levels after diets (21.46 ± 10.56)

At baseline, all our patients presented with high ECP levels compared with the normally accepted standard [6], with average levels of 34.253 μg/mL ± 26.3725. We first analysed changes in ECP levels for the 19 cases initially included in the study without correction for possible diet transgressions or blood samples taken in a seasonal period, and we observed a non-statistically significant reduction in ECP levels to 23.663 (μg/mL) ± 14.692 (*p* = 0.052). However, when we included only the 16 patients who followed the protocols regarding dietary transgressions and blood sampling without respiratory symptoms, we observed a pre-diet ECP mean of 37.3875 ± 26.66871 and post-diet ECP mean of 21.4688 ± 10.56325, which are significantly different (*p* < 0.001) (Fig. 1).

### Clinical response

Fifteen of the 19 patients showed optimal responses to their specific diets and remained asymptomatic without pharmacological treatment. This response was assessed using the EoO-QOL-A (Adult eosinophilic oesophagitis quality of life questionnaire) [7]. Of the 4 remaining patients, empirical diets of 6 food items [8] were indicated in 2 patients, as sensitization was not established or they did not respond to specific diets; one of these patients did not respond to the diet, and the other could not maintain it. In both cases, these patients continued treatment with inhaled corticosteroids and proton-pump inhibitor (PPIs). The remaining two patients continued with PPIs alone. Two respondent patients required symptomatic treatment during the seasonal period and followed the specific diet for the rest of the year. One of the patients responding to the specific diet presented resolution of the mucosal banding observed in the first endoscopic study. Our intention is to repeat the endoscopic study with the remainder of the included patients.

## Discussion

This study shows that ECP may be a useful biomarker for monitoring EoE patients being treated with specific diets. We are aware that the small sample size does not allow us to extrapolate beyond the efficiency of the diets used.

Until now most of the studies previously published in reference to the use of ECP as a biomarker for monitoring patients on treatment with diets show consistent but insignificant decreases in ECP levels following diets. However, we should highlight other publications in which ECP and other markers have shown significant decreases in response to pharmacological treatment with corticosteroids or PPIs [9–11]. As discussed in these publications it is imperative to achieve an adequate control of our patients through the use of non-invasive techniques that can be used in normal clinical practice. In this sense the ECP seems to be a good marker of inflammation if the determinations are performed correctly.

EoE is a complex entity characterized by a variety of phenotypes. It predominantly affects men between 30 and 40 years old, and it is generally diagnosed at least three years after the start of symptoms involving sensitization to food and environmental aeroallergens [12]. However, it is becoming increasingly apparent that EoE may be an under-diagnosed entity with a late diagnosis. This delay highlights the importance of actively searching for patients with compatible symptoms, as early diagnosis is fundamental to avoiding the development of complications, such as impaction of the alimentary bolus. In our experience, doctors should suspect this condition if patients report episodes of choking or if one detects “atypical” adult food sensitizations such as milk, egg and gluten. The patient should then be referred to a digestive medicine specialist for endoscopy and biopsy to confirm the suspected diagnosis.

Recent studies [13] have further described the therapeutic management of patients with EoE. It is noteworthy that clinical and histological improvements can be achieved in 50.5 and 60.8% of patients, respectively, using PPIs alone. Treatment with topical steroids (particularly fluticasone and budesonide) is also efficient. Dietary treatment leads to improvement in patients, with 6-food empirical and elemental diets being more efficient than diets developed based on allergy test results. The efficiency of the latter diet type is quite variable according to the literature (from 30 to 70%) [14].

Therefore, there are a number of management and monitoring solutions available for EoE patients, which makes selecting the best option for each patient very important [15].

Although treatment with PPIs or topical corticosteroids allows for good control of patients, life-long dependency on this type of treatment is not desirable. Establishing a diet that allows patients to control their condition in the long term without depending on chronic treatment is preferable. As previously discussed, the phenotypic variability of EoE makes it impossible to formulate simple treatment rules. A profile must be established for each patient with esophagitis, and the treatments must be adapted in each case. Based on our experience, specific diets were most widely accepted by patients, as they are easier to follow than the elemental (unfeasible in adults) and 6-food empirical diets. Unfortunately, this approach is not always possible. In cases in which we were able to establish diets with clinical responses and a decrease in ECP levels, we are sequentially reintroducing the foods in question (initially gluten or cooked legumes). We observed responses in each case and assessed the possibility of repeating the endoscopic study after a certain period of time to assess the response and concordance between histology, clinical improvement and ECP reduction.

## Conclusions

We believe that the serial determination of ECP levels is useful for monitoring most patients with EoE, given one avoids confounding factors such as infections, acute atopic dermatitis, dietary transgressions or respiratory symptoms, although we are aware that perennial symptoms may hinder the utility of this biomarker in many cases. More studies and batteries with more patients are needed to better understand this pathology.

### Study limitations

Our study has two important limitations: the small number of patients and the absence of a histological study after the establishment of the diets and ECP measurement. We are currently including more patients, although this is complicated by the low prevalence of this disease. Although an anatomo-pathological study is desirable as a gold standard, it is ethically questionable to subject an asymptomatic patient to an invasive technique with potential complications. We must remember that most of our patients remain asymptomatic without pharmacological treatment.

## Abbreviations

ECP: Eosinophil cationic protein
EoE: Eosinophilic esophagitis
PPI: Proton pump inhibitor
